# Current State of Professionalism Curriculum in Oral Health Education

**DOI:** 10.1111/eje.13048

**Published:** 2024-11-06

**Authors:** Melanie Nasseripour, Andreas Agouropoulos, Maria Theresa Van Harten, Maria Correia, Nibal Sabri, Annemiek Rollman

**Affiliations:** ^1^ Faculty of Dentistry, Oral and Cranio‐Facial Sciences, Centre for Oral, Clinical & Translational Sciences King's College London London UK; ^2^ School of Dentistry, National and Kapodistrian University of Athens Athens Greece; ^3^ Dublin Dental University Hospital Dublin Ireland; ^4^ Universidade Católica Portuguesa Faculty of Dental Medicine, Center for Interdisciplinary Research in Health Viseu Portugal; ^5^ University of Dundee Dunde UK; ^6^ Faculty of Dentistry, ACTA Amsterdam Netherlands

**Keywords:** curriculum, ethics, literature review, professionalism, survey

## Abstract

**Aim:**

The aim of this study was to systematically review the relevant literature on teaching professionalism in dental curricula and report the relevant data from a survey of members of the Association for Dental Education in Europe (ADEE) on the topic.

**Materials and Methods:**

We conducted a systematic review of the literature and a cross‐sectional study using an online questionnaire regarding teaching and assessment of professionalism in dental schools, members of the ADEE. The literature review identified 33 articles related to professionalism in dental curricula. The survey sent to the membership of the Association for Dental Education in Europe yielded responses from 27 European dental schools and four schools outside Europe.

**Results:**

Various study designs were identified in the review, and the main themes discussed included teaching/pedagogy, ethics/humanities, core domains and assessment of professionalism. Twenty schools reported having specific courses dedicated to professionalism, while the format varied, including entire modules, separate learning outcomes or unit structures. Ethics, communication, law/legal framework/deontology, teamworking and management/leadership were among the common topics taught under the banner of professionalism. Many schools reported increases or no changes in hours, staffing, themes/topics covered and weight in the curriculum for professionalism over the last decade.

**Conclusion:**

The paper provides valuable insights into the current state of professionalism education in dental curricula and offers directions for enhancing its effectiveness and relevance in preparing future dental professionals. Collaborative efforts among dental educators and institutions can contribute to the continuous improvement of professionalism education and practice in dentistry.

## 1 Introduction

In the dental education, the teaching and assessing professionalism present a multifaceted and complex challenge [1]. The intricacies stem from the diverse dimensions of professionalism, which extend beyond medical knowledge and technical skills to include interpersonal abilities, communication, cultural competence, ethical decision‐making and collaborative teamwork. The dynamic nature of professionalism further complicates the educational process [2]. Assessing professionalism introduces another layer of complexity, as traditional methods may not effectively capture the subjective and context‐dependent aspects of professional behaviour. The sheer magnitude of variables, such as individual differences, cultural shifts and institutional climates, adds more diversity to the assessment process [3].

Within the sphere of dental education, the imperative for a comprehensive definition and grasp of professionalism is accentuated, bolstered by thematic network projects initiated by the European Union (DentEd, DentedEvolves, Dented III, DentCPD) [4].

These projects are strategically designed to harmonise dental education, facilitating the mobility of healthcare professionals and patients within an expanding sector and market. Robust support for research, particularly focusing on competencies and training skills, underscores the commitment to elevating dental education standards. Aligned with these thematic endeavours, experts from the Association for Dental Education in Europe (ADEE) have actively engaged in advancing recent developments in European dental education. Notably, the 2022 ADEE general assembly conference held in Palma De Mallorca featured dedicated sessions aimed at delving into the nuanced understanding of professionalism.

Recognising the complexity and the multidimensionality of professionalism is essential for developing effective strategies that prepare future healthcare professionals for the evolving landscape of dental practice. It is evident that there is lack of consensus within the dental education community about the teaching and evaluation of the dental students regarding this issue [5]. Nevertheless, in the modern world where mobility of dental professionals between European countries and internationally is very common, it is important to have a shared understanding of the issue and provide a toolkit to assist dental schools to incorporate professionalism teaching and evaluation in their curricula [1]. In response to this identified gap, ADEE took a proactive stance, channelling efforts and research towards the maturation of professionalism among young dental students. This initiative led to the establishment and support of a Community of Practice (COP), fostering collaboration and knowledge exchange in various forums, including virtual meetings as well as physical congresses especially at ADEE 2023 conference held in Liverpool. This collective effort signifies a commitment to bridging the divide and establishing a unified framework for professionalism in dental education. As a result of the ADEE Professionalism COP discussion, a study was conducted revolving around the conceptualisation of professionalism, examined through the lenses of ethical guidelines and educational approaches within the context of dental and para‐dental education. The aim of this study was to systematically review the relevant literature on teaching professionalism in dental curricula and report the relevant data from a survey of members of the ADEE on the topic.

## 2 Materials and Methods

The first part of the study was a systematic review of the literature, and the second part was a cross‐sectional study using an online questionnaire regarding teaching and assessment of professionalism in dental schools, members of the ADEE. The study was approved by the Board of the ADEE.

**FIGURE 1:**
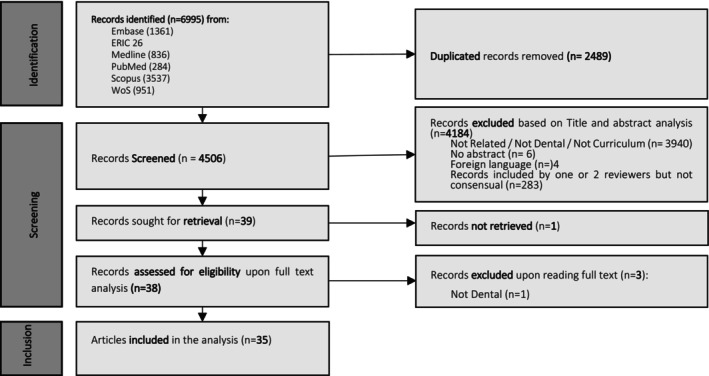
Flow chart of the selection of articles for the literature review.

The systematic review was conducted following the Preferred Reporting Items for Systematic reviews and Meta‐analysis (PRISMA) guidelines. The question to be answered was: ‘Are there recommendations/evidence to be included in curriculum for the teaching and assessment of professionalism in dentistry science?’ An extensive systematic literature search was conducted using the following query: (dent* or odontology) AND ((professionalism) OR (ethics) OR (deontology)). Filters used were English language and time from 2010 to 2023. We looked at articles from 2010 onwards as many curriculum and guidance documents are updated frequently and we were interested in the most recent and we also noted that challenges posed by professionalism teaching and assessment have changed rapidly and the authors decided to focus on currently relevant aspects of professionalism teaching. From the 6995 records identified in the databases searched (Embase, ERIC, Medline, Pubmed, Scopus and Web of Science), 2489 duplicated records were removed and 4506 were screened by reading of title and abstract. Criteria for inclusion were articles looking at professionalism in the dental curriculum. Articles which referred to other health‐related professions, which focused on assessment of professionalism, or which were not curriculum related were excluded as referred in Figure 1. Three independent researchers (AA, MN and MJC) screened the 4506 articles, and 39 articles were included by all three reviewers and therefore were selected for full text analysis. From the 39 eligible articles, one was not possible to retrieve, and three were further excluded either because they were not related to dental students [1] or because they were not curriculum related [2].

A total of 35 articles were screened as to their quality using the Joana Briggs Institute tools for qualitative research appraisal tools [6, 7, 8]. A critical appraisal was conducted by three researchers (AA, MN and MJC) independently, consensus was reached when all three appraisers agreed. Results from the critical appraisal are summarised in Table 1. Quality analysis and data extraction were performed by four different authors (AA, MN, MJC and NS) separately and in cases where there was disagreement, a consensus position was achieved after considering the analysis of the four authors.

**TABLE 1:** Main characteristics of the studies included in the review and critical appraisal.

	Author and year	Country	Study design	Participant type	Main themes	Critical appraisal
1	Neville et al. 2018 [9]	United Kingdom	Mixed methods	Dental students (undergraduate)	Dental scrub ceremony	Moderate
2	Chuenjitwongs et al. 2018 [10]	Tailand	Mixed methods	Dental students (undergraduate) and dental educators	Core domains	High
3	Smith et al. 2018 [11]	New Zealand	Qualitative	Dental educators	Teaching/Pedagogy	Moderate
4	Cino et al. 2018 [12]	USA	Cross‐sectional	Dental students (undergraduate)	Ethics and humanities	High
5	Raman and Ramlogan 2020 [13]	Jamaica	Mixed methods	Dental students (undergraduate)	Ethics and humanities	High
6	Tenenbaum et al. 2020 [14]	France	Mixed methods	Dental students (undergraduate)	Ethics and humanities	High
7	Calleja et al. 2020 [15]	Chile	Review	N/A	Ethics and humanities	Moderate
8	Quick and Blue 2019 [16]	USA	Mixed methods	Dental students (undergraduate)	Teaching/Pedagogy	High
9	Rosa et al. 2022 [17]	Indonesia	Qualitative	Dental students (undergraduate) and dental educators	Teaching/Pedagogy	Moderate
10	Ahmad et al. 2020 [18]	Malaysia	Qualitative	Dental students (undergraduate) and dental educators	Teaching/Pedagogy	Moderate
11	Ranauta et al. 2018 [19]	United Kingdom	Qualitative	Dental students (undergraduate)	Teaching/Pedagogy	High
12	Sarabadani et al. 2022 [20]	IRAN	Cross‐sectional	Dental students (undergraduate)	Assesment of professionalism	High
13	Noushi et al. 2020 [21]	Canada	Review	N/A	Core domains	High
14	Seminario et al. 2020 [22]	USA	Cross‐sectional	Dental students (undergraduate and postgraduate)	Teaching/Pedagogy	High
15	Jongpipittaporn et al. 2022 [23]	Thailand	Qualitative	Dental students (postgraduate)	Teaching/Pedagogy	High
16	Ergunol et al. 2022 [24]	Cyprus	Cross‐sectional	Dental students (undergraduate)	Teaching/Pedagogy	Low
17	Neville 2018 [9]	United Kingdom	Descriptive report/opinion	Dental students (undergraduate)	Teaching/Pedagogy	High
18	Bukusi 2022 [25]	Kenya	Qualitative	Medical students (undergraduate)	Teaching/Pedagogy	Moderate
19	Hanks et al. 2022 [1]	United Kingdom	Opinion	N/A	Core domains	High
20	Mather et al. 2022 [26]	United Kingdom	Mapping study	N/A	Teaching/Pedagogy	High
21	Shah et al. 2022 [27]	Canada	Cross‐sectional	Dental students (undergraduate)	Core domains	Moderate
22	Kwon et al. 2022 [28]	Canada	Qualitative study	Dental students (undergraduate)	Core domains	Moderate
23	Imtiaz et al. 2022 [29]	Pakistan	Qualitative	Dental patients	Ethics and humanities	Moderate
24	Goetz et al. 2021 [30]	Germany	Cross‐sectional	Dental students (undergraduate) and dental educators	Ethics and humanities	High
25	Ali 2021 [13]	Pakistan	Cross‐sectional	N/A	Teaching/Pedagogy	Low
26	Partido 2020 [31]	USA	Cross‐sectional	Dental hygiene students (undergraduate)	Ethics and humanities	High
27	Ahmedani 2020 [32]	Saudi Arabia	Cross‐sectional	Recent graduates and interns	Core domains	High
28	Friedlander 2019 [33]	New Zealand	Descriptive report	Undergraduate curriculum	Teaching/Pedagogy	High
29	Bateman 2019 [34]	United Kingdom	Opinion	N/A	Core domains	High
30	Alcota 2019 [35]	Chile	Cross‐sectional	Dental students (undergraduate) and dental educators	Ethics and humanities	High
31	Hertrampf 2018 [36]	Germany	Qualitative	Dental students (undergraduate) and dental educators	Ethics and humanities	Moderate
32	Bateman 2018 [37]	United Kingdom	Document analysis	N/A	Teaching/Pedagogy	High
33	Holden 2018 [38]	Australia	Opinion	NA	Teaching/Pedagogy	Moderate

The second part of this study was a cross‐sectional study using an online questionnaire exploring how professionalism is taught and assessed in dental schools; specifically, ADEE member schools. Questions for the survey were created by dental educators from across Europe involved in the ADEE Professionalism Community of Practice. Schools were encouraged to participate in the Qualtrics‐hosted survey via the monthly newsletters distributed to ADEE members. The survey was open from 17 May 2023 to 20 Sept 2023. Data from schools that did not fully complete survey were excluded. Microsoft Excel and IBM SPSS were used to manage the quantitative data and generate descriptive statistics. Qualitative responses to open‐ended questions were summarised.

## 3 Results

### 3.1 Literature Review

**FIGURE 2:**
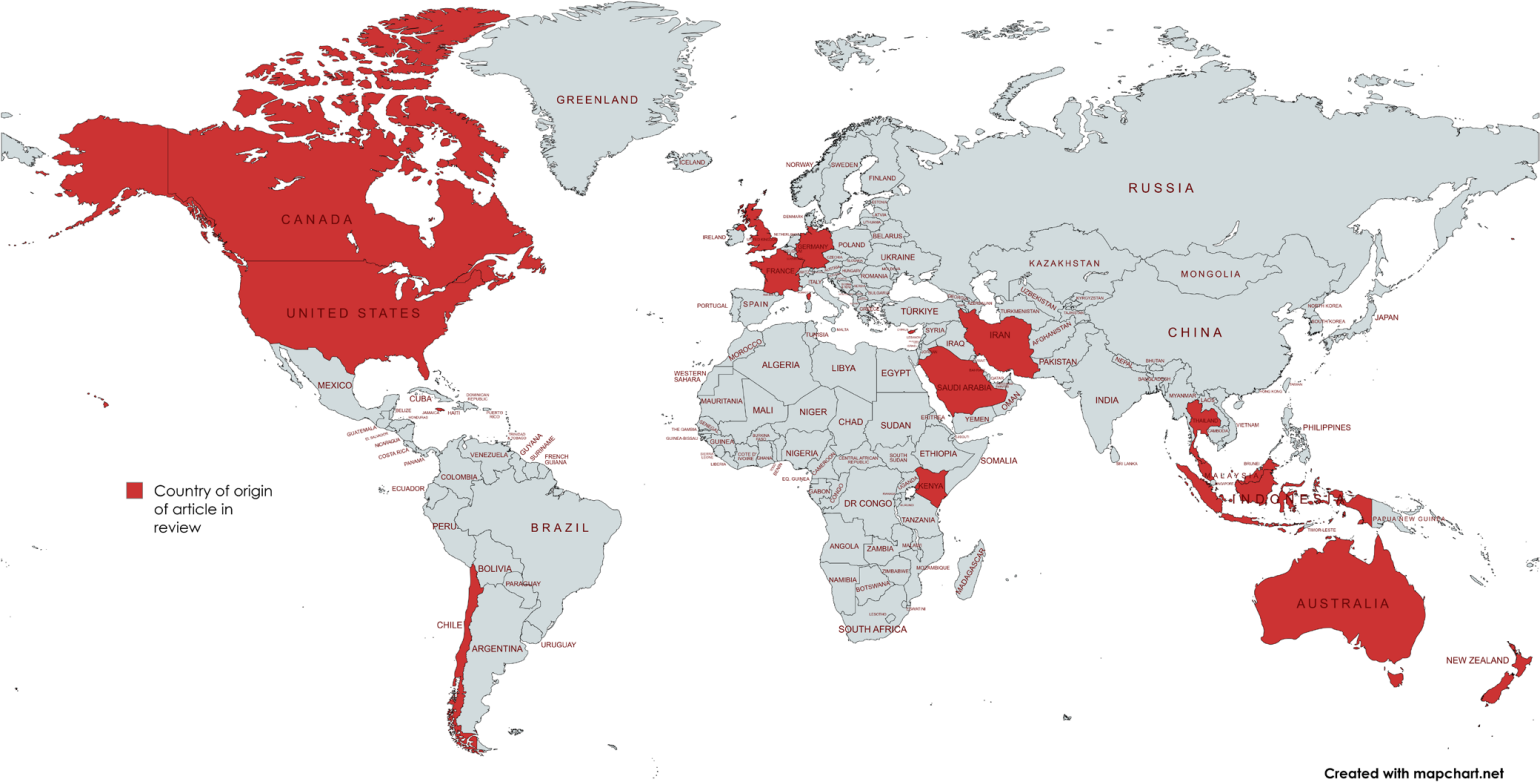
The countries of origin of the articles included in the review.

Figure 1 presents the flow chart of the review process that reached to 35 articles which were evaluated for quality. Two articles were excluded because one was considered unclear and one was a comment on another article, so finally 33 articles were included in the review. Figure 2 presents the geographical spread of included articles. Although the United Kingdom (*N* = 7), United States (*N* = 4) and Canada (*N* = 3) were the countries with most of the included reports, there were articles from all areas of the world. Regarding types of studies, (shown in Table 1), there was a wide variation with most of them being qualitative (*N* = 10), cross‐sectional studies (*N* = 9) and mixed methods (*N* = 5), while there were descriptive reports (*N* = 3), opinion papers (*N* = 3), reviews (*N* = 2) and one mapping study. Most of the original research papers included dental/medical students (*N* = 22), dental educators (*N* = 6), patients (*N* = 1) and recent graduates/interns (*N* = 1). There was a great variation of the main themes discussed in the papers: teaching/pedagogy (*N* = 14), ethics/humanities (*N* = 9), core domains (*N* = 7), ceremony (*N* = 1) and assessment (*N* = 1). Subthemes were authentic teaching, role modelling, cultural competence, reflection and definition of professionalism.

### 3.2 Survey

**FIGURE 3:**
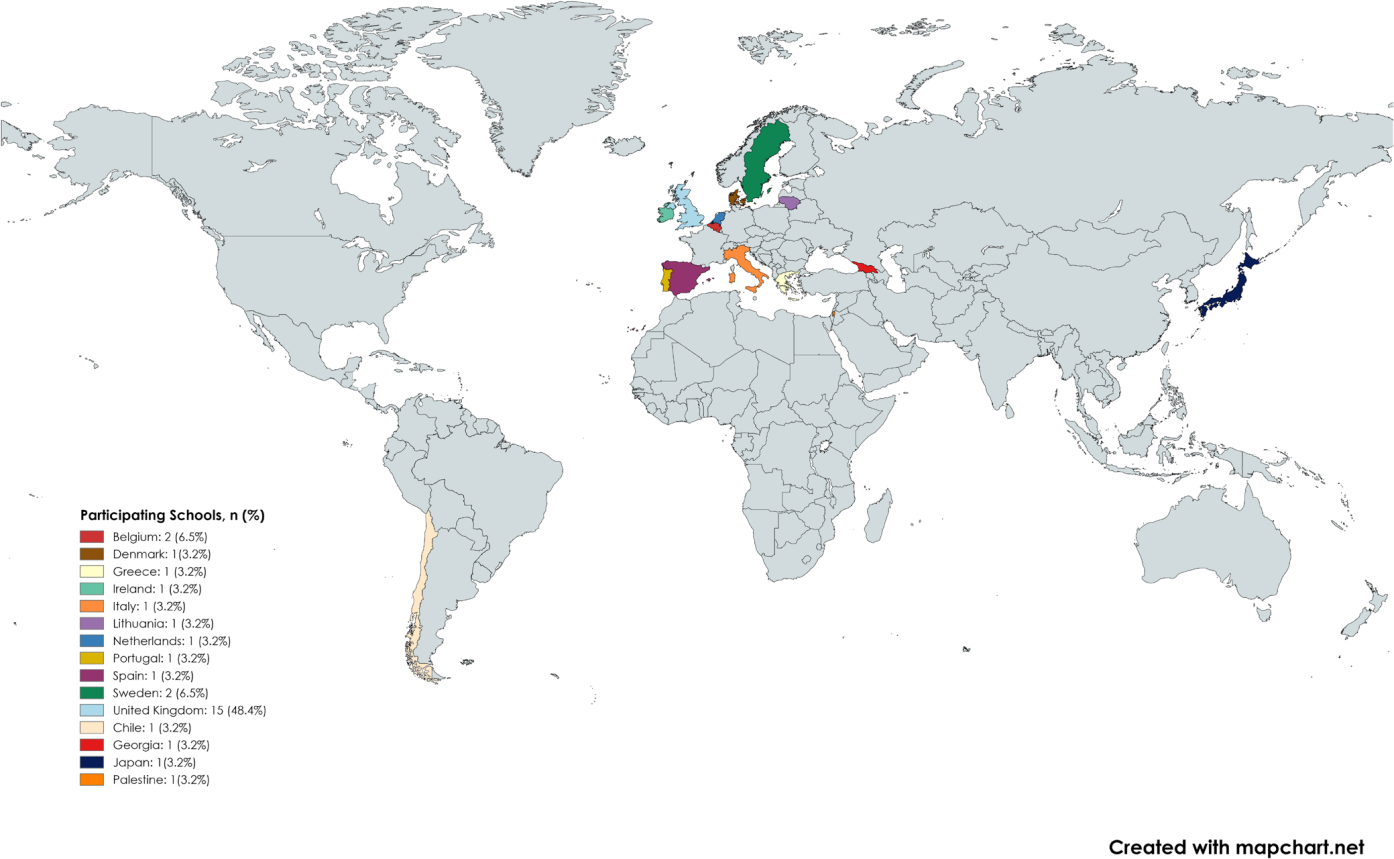
Countries and number of Dental Schools per country participated in the survey.

The invitation to participate in this survey was sent by newsletter to the membership of the Association for Dental Education in Europe: 135 European dental schools with full membership and 40 schools outside of Europe, plus dental and hygienist associations. Of the 39 schools attempting the survey, only 31 completed the survey in full; thus, these are the responses presented herein. Twenty‐seven schools that responded are European, while four are not (Figure 3). The majority of schools that responded offer Dental Undergraduate programmes (*n* = 28). Many offer Dental Post Graduate Studies also (*n* = 23). Fewer offer Dental Hygiene (*n* = 16) and Dental Therapy programmes (*n* = 11).

Twenty schools reported having specific course(s) dedicated to professionalism; while nine did not; and two responded with ‘other’, and explained that aspects of professionalism are incorporated into the curriculum but are not collectively named as a professionalism course. When asked what format is used for teaching professionalism, schools reported that it is taught as an entire module (*n* = 8) or unit (*n* = 2). For many schools, professionalism is structured as a collection of separate learning outcomes mapped to multiple and/or different units and modules (*n* = 11) (Table 2). Indeed, some respondents in open text described professionalism as being ‘embedded into other modules over the course of the programme’, being ‘structured as non‐modular’, ‘a spiral curriculum with several themes’ taught across all years. Further, the most common response to the survey question asking when professionalism is taught, was all throughout the programme (*n* = 23). In response to whether their professionalism course, unit or module was mandatory or compulsory learning for students, 28 responded that it was (Table 2). The school that responded that the professionalism component is not mandatory, explained that indeed some of the content within professionalism is compulsory but some is not. Some schools in open text mentioned that students have very few electives. Others mentioned professionalism as necessary for progression. Table 2 presents the topics taught under the banner of Professionalism with Ethics and Communication being the most common. In open text, some schools specified additional topics that are covered. These were as follows: Equality and Diversity and Inclusion/Sustainability, Life philosophy, Self‐management, Dental entrepreneurship, Continuing professional development, Feedback, Clinical governance and regulation.

**TABLE 2:** Characteristics of the format, timing and content of professionalism in the institutions participating in this survey.

	Count (*n*)	Per cent (%)
*Format*		
Collection of separate learning outcomes mapped to multiple/different units/modules	11	51.6
Entire module	8	25.8
Other	4	12.9
Unit within a module	2	6.5
*Timing*		
All through the programme (i.e., from first to last year)	23	74.2
Multiple years	5	16.1
Specific year	3	9.7
*Mandatory/compulsory*		
Yes	28	90.3
No	1	3.4
Other	2	6.5
*Topics taught under the banner of professionalism*		
Ethics	30	96.8
Communication	28	90.3
Teamworking	24	77.4
Law/legal framework/deonotology	26	83.9
Management and leadership	18	58.1
Practice management	18	58.1
Social behaviour sciences (sociology/psychology)	15	48.4
Public health/dental public health	15	48.4
Health systems	12	38.7
Other	3	9.7

**FIGURE 4:**
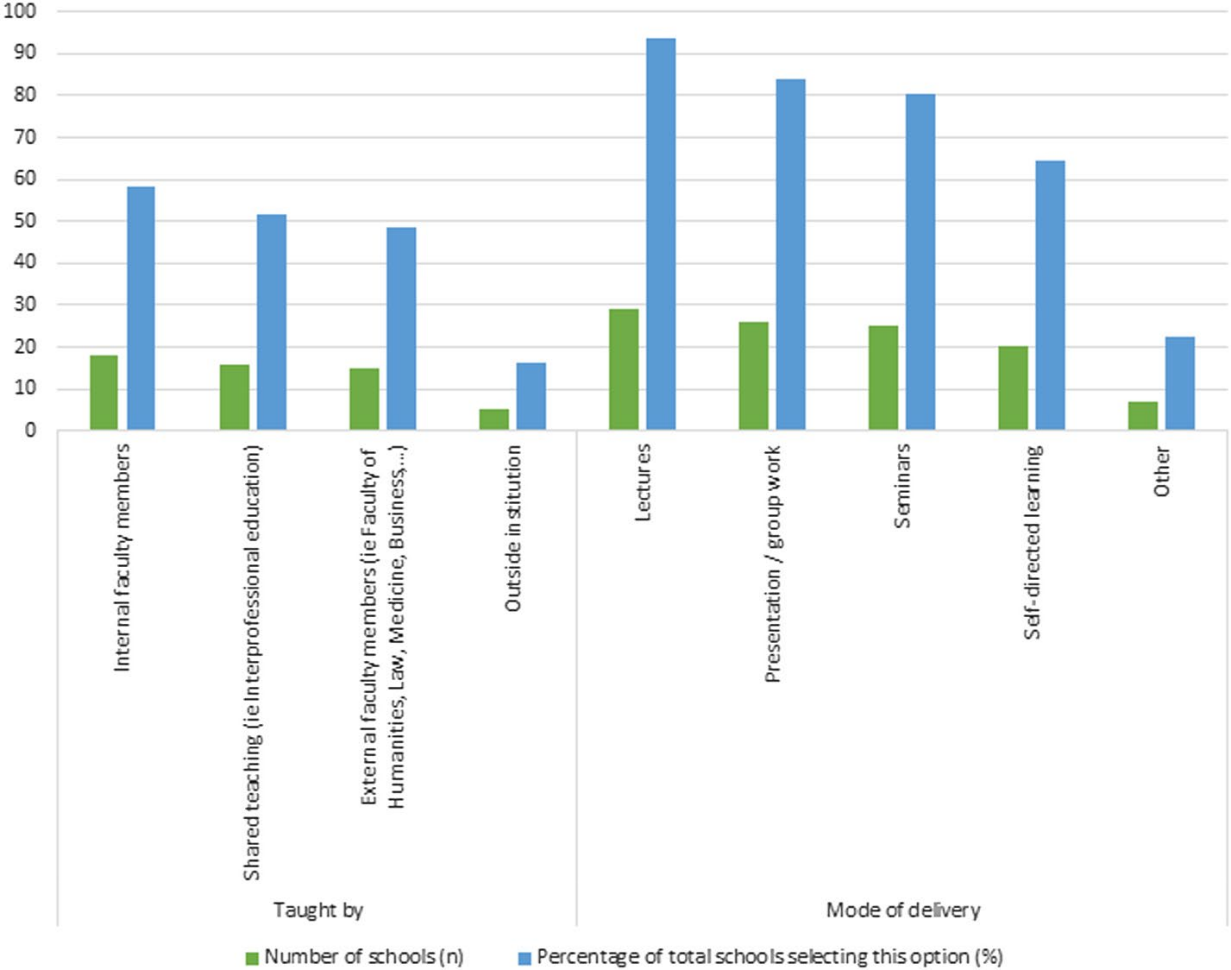
The delivery of professionalism topics in participating schools.

In many schools that responded, professionalism is only taught within each programme by internal faculty members (*n* = 18) (Figure 4). Some schools reported professionalism as taught by external faculty members (*n* = 15). And less frequently, it is taught by an outside institution (*n* = 5). Often, schools reported that teaching of professionalism is shared, meaning interprofessional education (*n* = 16). Most schools reported that professionalism content is delivered by lectures (*n* = 29). Presentation/group work, seminars and self‐directed learning modes are also popular. In open text, schools included other modes such as volunteering; groups of students roleplaying with recording and playback; posters and online blogs; multi‐modal means; practical training; and mixed delivery—in‐person, asynchronous, live online.

**FIGURE 5:**
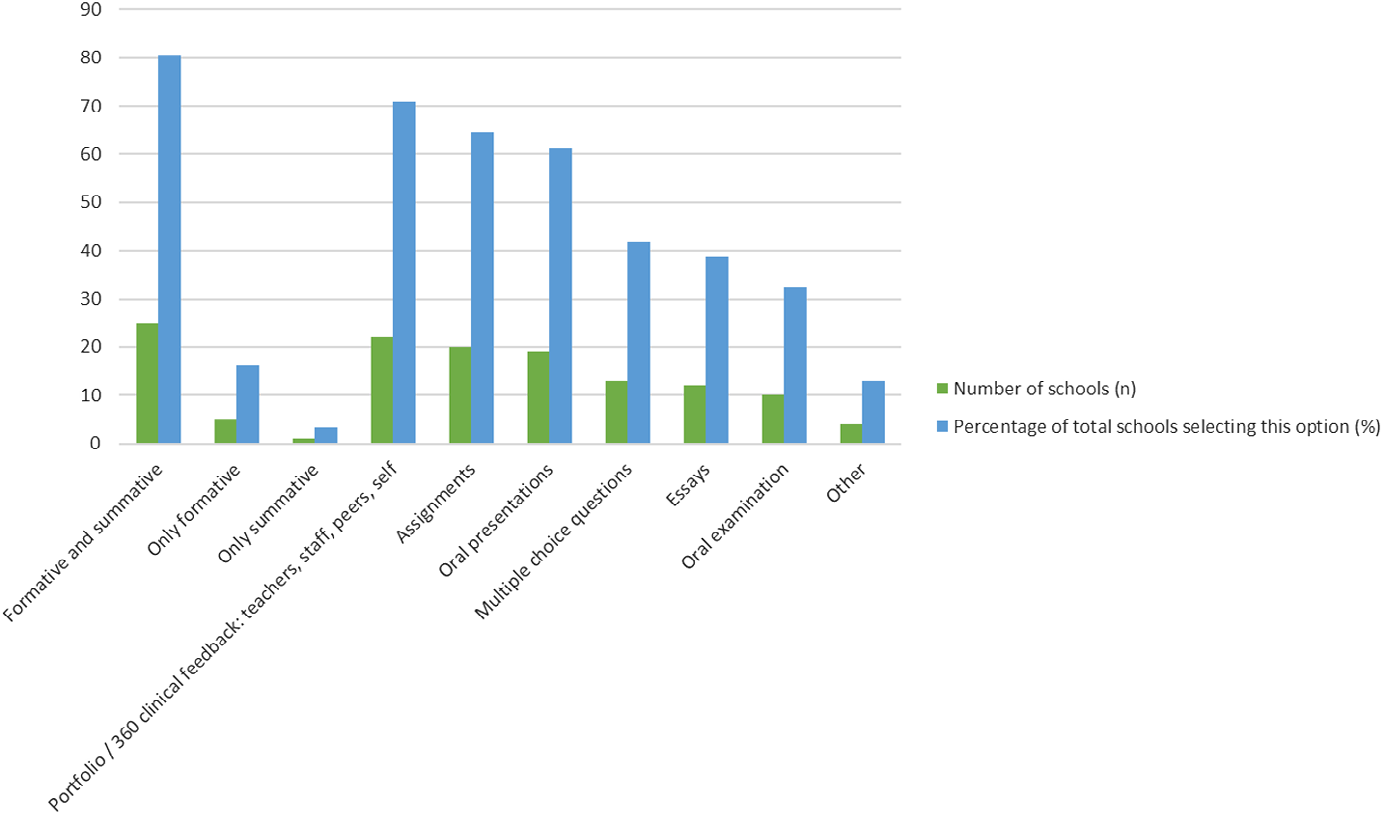
Assessment type and methods associated with professionalism.

Twenty‐five schools report assessing students on Professionalism using formative and summative assessment (Figure 5). Fewer than 10 responded that they only use one or the other. The most common mode of assessment was through Portfolio/360/clinical feedback from teachers, staff, peers and self. But Assignments (*n* = 25) and Oral Presentations (*n* = 24) are also very common. Many schools also assess Professionalism through Essays, Multiple choice questions, Oral Examination and other means.

Consistent with the variability of responses to the survey questions about format and timing of Professionalism teaching, responses asking about the ECTS credit value of Professionalism teaching also revealed wide variability. Some schools did not appear able to quantify Professionalism teaching by ECTS credits and format: either they selected the does not apply option or did not select an option at all. Two schools responded that their Professionalism course is worth 10 ECTSs, six schools have a Professionalism module ranging from 10 to 60 ECTSs, two schools have a separate Professionalism learning outcome worth 15 and 30 ECTSs and four schools have an alternative format which is worth 10–30 ECTSs.

**FIGURE 6:**
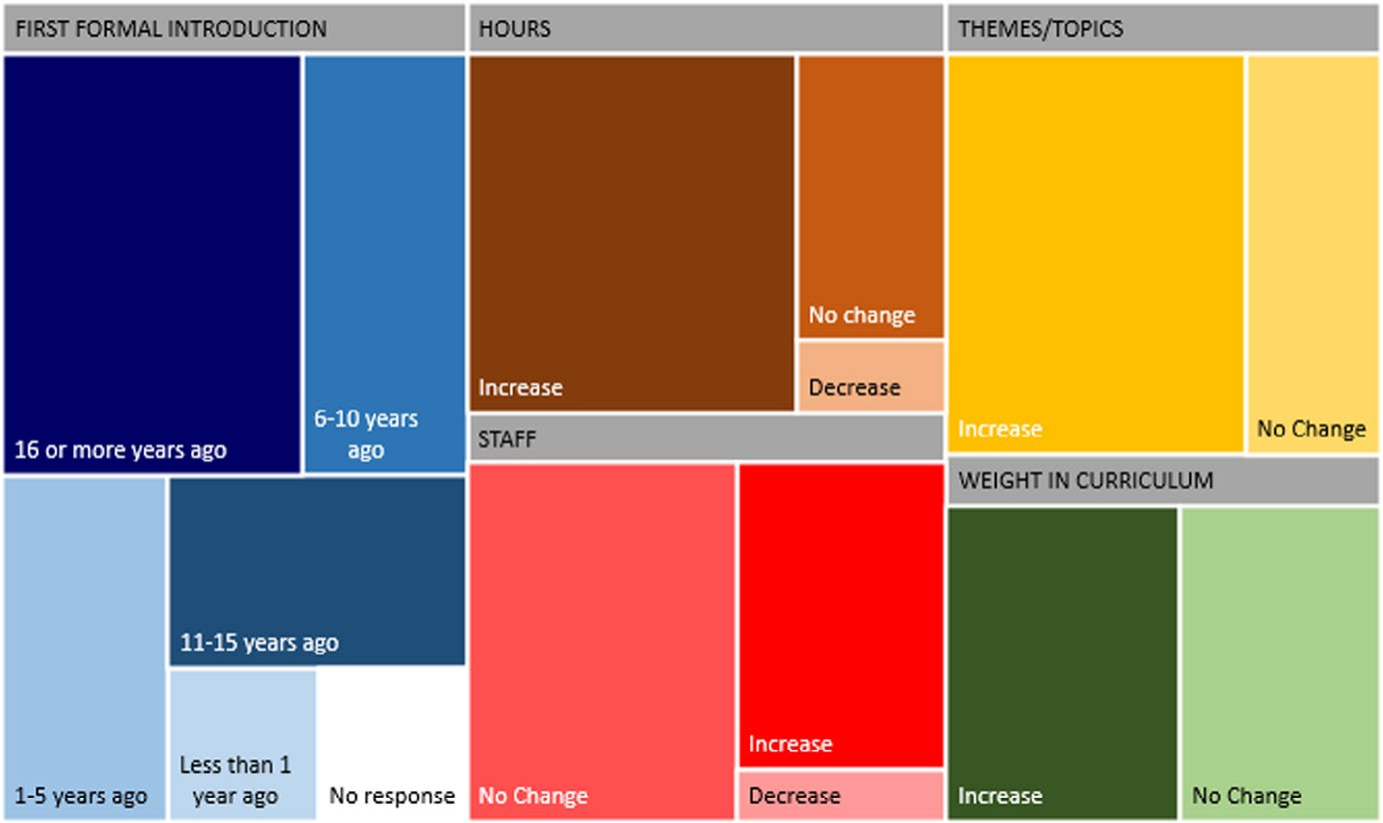
Teaching of professionalism over time: Introduction and Changes in the Last Decade at Institutions teaching Professionalism 11 years or longer.

Sixteen of the 36 schools who responded specifically to the question of how long teaching Professionalism has been part of the curriculum, stated that it has been 11 or more years since it was formally introduced (Figure 6). Most of these schools report either an increase or no change in hours, staffing, themes or topics covered and weight in the curriculum for Professionalism over the last decade. Few reported decreases in these aspects.

**FIGURE 7:**
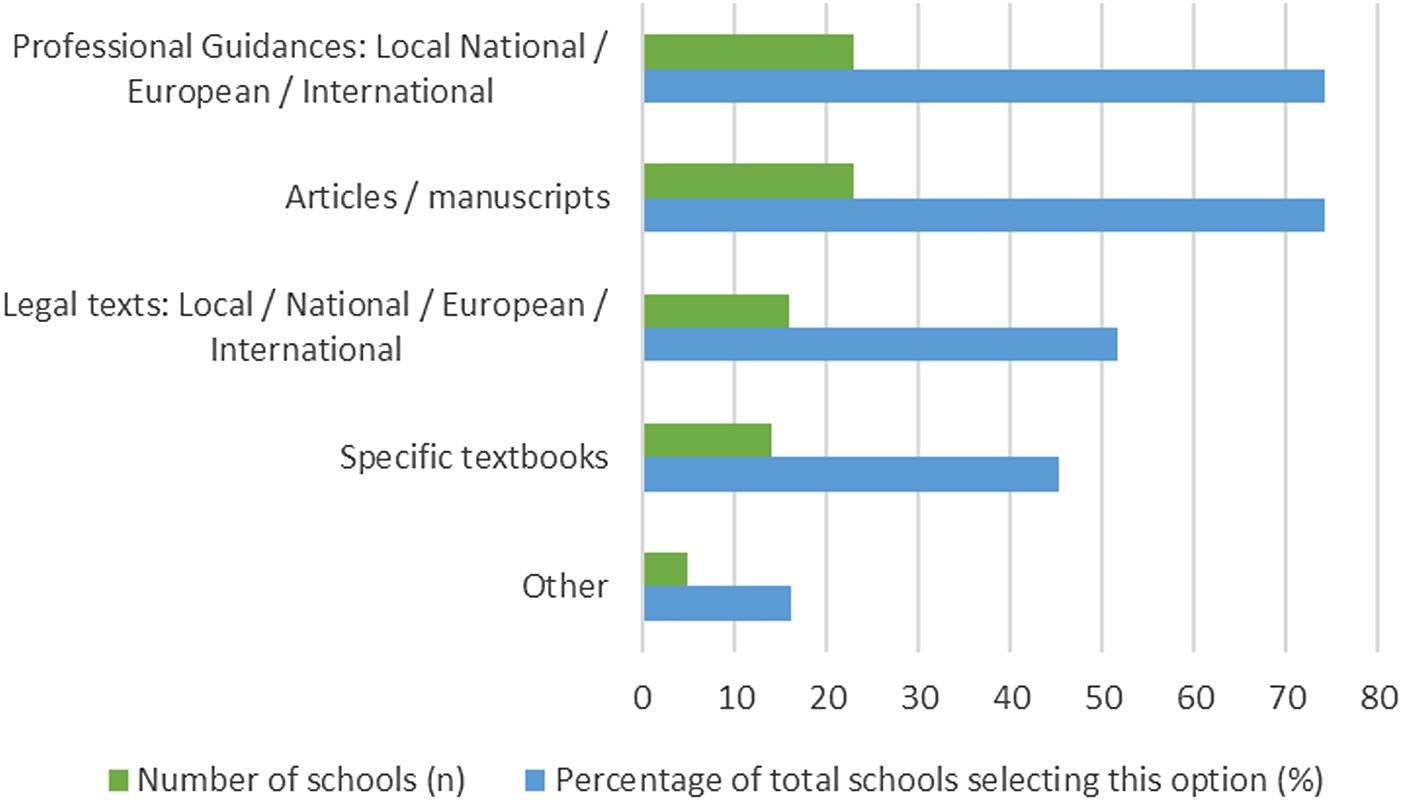
Resources and materials used in teaching professionalism.

A variety of different resources and materials are used in teaching Professionalism (Figure 7). These include Professional guidance, articles, manuscripts, legal texts and specific textbooks. In at least two schools, the respondent was unsure what was provided, as it was the responsibility of the individual educator to provide these.

When asked for suggestions regarding the ADEE Professionalism CoP's role regarding Professionalism curriculum, many comments focused on sharing experiences, and streamlining curriculum and teaching materials. Schools thought that the ADEE CoP might be of assistance in helping to establish a professional ‘student’ attitude, provide guidance in dealing with lapses in professionalism and suggest ways to improve cultural competency and inclusive practice.

## 4 Discussion

As there is still no consensus on a set definition of the term ‘professionalism’ in oral healthcare, this leads us to discuss/debate what the concept ‘looks like’ in oral healthcare [11]. When attempting to create definition, you must look at a shared definition from multiple stakeholders (public/patients/professionals/ regulators/students) [26]. It is also essential to have this definition as professionalism has become an important aspect of the undergraduate learning outcomes for dental students and is essential to grasp it if we are to tackle the definition of the safe practitioner [27].

There are four core domains essential for teaching roles and in which all educators should be competent: (1) ‘Educational principles’, (2) ‘educational practice in dentistry’, (3) ‘Curriculum, quality, and improvement’, (4) ‘Educational professionalism’ [10]. In essence, there has been an increase in the number of outcomes graduates must attain before they can graduate, with professionalism weighing in quite significantly and ever so increasing in its weight [27]. The review showed that ethics, ethical constructs and ethical context should be incorporated and flagged as a competency [30], which is verified by the survey, where ethics was the most frequent answer provided by the schools.

Professionalism is unique in that it can be associated with all other disciplines. Interprofessional education can increase self‐efficacy after collaborating with students from different health professions programmes [12]. It should therefore be embedded in every aspect of teaching, learning and practice [11]. The survey results showed that in most schools, professionalism is taught throughout the curriculum. There is a statistically significant difference between integrity/responsibility/respect and service‐mindedness among dental students of different years and one must consider a vertical and horizontal integration of the teaching and learning of professionals in the undergraduate curriculum [20, 39]. In addition, the curriculum must consider formal teaching in communication as it is often either not taught or not taught well, and this is compounded by the lack of senior role models to show how to communicate well [1], which was not found in the survey as part of the teaching modalities on professionalism.

Most oral health teachers come directly from clinical practice and therefore often have limited pedagogical knowledge and experience [11]. Students' positive transformative learning will require not only changing the dental school climate, curriculum but also faculty training, with a school wide commitment to supporting professionalism and reflective practice of students and educators. This involves, in addition to staff training and support, adequate resources and clear school guidelines since positive role modelling and personal reflections have been suggested as the most effective elements for the development of professionalism in teaching programmes [1, 21, 25]. The responses regarding the teachers, showed that it is not only the dental faculty involved in the teaching of professionalism but also external faculty members. This probably confirms lack of skills and maybe willingness by the dental faculty members to participate in teaching of professionalism, requiring external support. External faculty could be academicians or other dental professionals (clinical instructors, researchers, etc.) from the areas of ethics/humanities or outside practice bringing a different perspective to the dental studies and other dental professionals providing a more diverse insight of the issue.

In the medical literature, an official white coat ceremony, is widely reported as part of the development of a professional identity by the medical students. In this review, only one paper referred to that topic indicating that this is not a frequent practice for the dental schools [40]. The introduction of an honour code with or without an official ceremony when students get clinical privileges is considered to provide an opportunity to reflect on their responsibilities as dental students, reminding them of the vocational aspect of their work, and the role of trust and respect in professionalism [39]. It can be coupled with creative pedagogical strategies, help to foster understanding and appreciation for professional (including academic) integrity [41]. Nevertheless, the survey showed that usually, professionalism issues are taught by traditional methods (lecturers, seminars, projects etc.) and official ceremonies are not part of the professionalism teaching.

The best way in which institutions can promote professionalism is by role‐modelling it themselves (professional role models) as in real clinical settings, which generates strong emotions [11, 19, 39]. Enabling good mentorship and providing resources educators can support producing the next generation of not just competent but compassionate young practitioners [1]. Experiencing unprofessional faculty behaviour can impact students who may then consider that if this is acceptable behaviour for faculty members, then it must be acceptable for students [28]. Role modelling and mentoring is considered as one of the five key components influencing Professional Identity Formation alongside domain‐specific self‐efficacy, professional socialisation with peers, learning environment and reflection [29].

It is advisable to incorporate simulation techniques as part of the teaching and evaluation of clinical ethics and not isolated to classroom teaching [1, 9, 15]. These have been reported in the survey by a small number of institutions, which shows the need by bodies like ADEE, to provide toolkits to the dental schools for improving the teaching of professionalism.

The social interactions with peers and outside practitioners facilitate introspection and growth [16] and experiences within clinical environments strongly influence students' understanding of professionalism [19]. This exposes students to the concept of humanised patient‐centred care paradigm [24]. Professional socialisation with peers and learning environment are basic key components influencing professional identity formation.

As mentioned above, ethics was the most frequently reported topic, taught in the professionalism courses from the survey responders, but in the review, a clear need for more integration of humanities within dental curricula was identified. For example, narrative medicine and its methodology can support students' focus on hearing the patient voices when they are articulating their experience of ill‐health. A collaborative approach would have a powerful effect on dental students' development as humanistic and empathetic practitioners, while ensuring the satisfaction of clinical and professional competencies [22].

Ethics and social responsibility teaching could offer a way to turn positive attitudes into real competencies and should be considered at an early stage [1, 21, 35]. But these subjects must not only be isolated to classroom lectures but also explored in hands‐on discussions of scenarios [9, 13], to identify, solve or reflect on ethical problems and incorporating simulation techniques [15]. It is also suggested that the patients' ethical constructs and ethical context may be incorporated into the dental ethics course [30].

An important recommendation is to consider the use of innovative learning methods from local cultural materials to improve the empathic communication skills of dental students and focus on cultural competency [17, 21, 24]. Furthermore, there must be room to include patients' cultural and ethical constructs [30]. Particular attention should be given to the patients' perspective, and their preferences which is essential but often overlooked. It should be noted that there seems to be a disparity in the understanding when the dentist tries to comprehend what the patient understands about ethics, with less educated patients believing in utility‐based ethics while more educated patients believe in universal and duty‐based ethics [29]. In general, dental patients stressed honesty, communication ethics, respect for patient and non‐maleficence and think some ethical attributes are desirable but they can be compromised, for example, autonomy, respect and trust, as the main topics regarding professional dental behaviour. Moreover, there seem to be different patients' ethical constructs in the middle east compared to the western world and implemented code of ethics, a fact that highlights the need for cultural respect by the dental professional [29]. To help broaden the students' perspective on oral health and differences in experience, there is recognition of the effectiveness of integrating global health within the regular dental curricula [23].

Since there is a lack of a definition of professionalism further, integral to teaching and fostering professionalism, is to determine how it can be assessed [28]. There is research gap on appraising attitudes towards social responsibility and social contracts and professional behaviour. The schools with official courses on professionalism, reported, in most of the cases, formative and summative assessments using a range of methods as presented in the survey results. Kwon et al., raised the question *that* ‘we end up looking at many metrics and doing a 360 evaluation taking multisource feedback, but how do we analyse all this data and what does it mean?’ implying that a more holistic approach is needed to ensure a proper, in depth evaluation of the topic [28]. This is probably the greatest challenge since it requires novel approaches and change of mind set.

It is imperative to cultivate through self‐reflection, mindfulness and social responsibility, as well as positive professional attitudes and behaviours [21]. This type of contemplative practice can have positive impacts on the competence development in both personal and professional perspectives [24, 25]. Indeed, reflection is another of the five key component influencing professional Identity formation.

The present report is based on previous studies with diverse focus on different topics of professionalism. While this is a limitation in systematic reviews, in the case of the studied topic is a strength since it covers most of the fields that ‘professionalism’ consists of. On the other hand, there are also limitations from the original articles, like sample sizes, representativeness of the selected samples, personal biases and diversity of the methodological approaches mainly in the qualitative papers. These are identified challenges that could be addressed in future research to provide more robust results.

Based on the key findings of the systematic review and survey conducted on the teaching and assessment of professionalism in dentistry, some directions for future improvements can be identified. There is a clear need for standardising and clarifying professionalism curricula across dental schools and incorporate interprofessional education and diverse teaching modalities to enrich professionalism education. Moreover, reviewing and adapting of professionalism curricula to reflect evolving societal expectations and professional standards is also important. Assessment methods should be enhanced to ensure comprehensive evaluation of professionalism traits and behaviours and establishment of collaborative platforms, such as the ADEE Professionalism CoP, for sharing best practices, resources and experiences in teaching professionalism could be extremely beneficial.

In conclusion, the systematic review and survey provided valuable insights into the current state of professionalism education in dental curricula and offered directions for enhancing its effectiveness and relevance in preparing future dental professionals. Collaborative efforts among dental educators and institutions can contribute to the continuous improvement of professionalism education and practice in dentistry.

## Author Contributions

All authors listed confirm they have contributed to the conception and design, or/and acquisition of data, or/and analysis and interpretation of data; and been involved in drafting the manuscript and revising it critically for important intellectual content; and given final approval of the version to be published. Agreed to be accountable for all aspects of the work.

## Ethics Statement

The work presented in this manuscript does not pertain to human studies and did not require ethical approval by institutions review boards/ethics committee.

## Conflicts of Interest

The authors declare no conflicts of interest.

## Data Availability

The data that support the findings of this study are available from the corresponding author upon reasonable request.
